# Is It Time for Sports and Health in the Era of Covid-19 Pandemic?

**DOI:** 10.3390/ijerph18020372

**Published:** 2021-01-06

**Authors:** Pantelis T. Nikolaidis, Beat Knechtle

**Affiliations:** 1 School of Health and Caring Sciences, University of West Attica, 12243 Athens, Greece; 2 Laboratory of Exercise Testing, Hellenic Air Force Academy, 13671 Acharnes, Greece; 3 Institute of Primary Care, University of Zurich, 8091 Zurich, Switzerland; beat.knechtle@hispeed.ch; 4 Medbase St. Gallen Am Vadianplatz, 9001 St. Gallen, Switzerland

When we took the initiative for this Special Issue, we were uncertain about its success. Would it get submissions? Would the submissions be of high quality? Hopefully, a large number of papers were published. These high-quality papers covered a wide range of topics in Sports and Health such as different ball games [1,2,3,4,5,6], training analyses [7,8], and health aspects such as vitamin D in adolescent athletes [9], treating obesity and the metabolic syndrome [10,11], infectious diseases such as HIV [12], exercise addiction [13], the level of mood and depression [14], tobacco use in elite athletes [15], and the aspect of the Covid-19 pandemic [16,17]. We hope that these papers will contribute to the advancement of Sports and Health sciences by offering practical applications for professionals in the field.

We recognize that the Covid-19 pandemic influences all aspects of sports activities worldwide. On the other hand, it poses new challenges for Sports and Health sciences. To counteract its negative impact on both physical and psychological characteristics, many humans started to exercise outdoors (e.g., running and cycling). Already, there was an increase in recreational athletes participating in sports competitions such as half-marathons [18], marathons [19] and ultramarathons [20,21,22,23] during the past decades. In both cases (start exercising either during the Covid-19 pandemic or before), the engagement of humans—often at an advanced age and without sports experience—in regular exercise with high training volume and/or intensity raises many questions for scientists in Sports and Health. For instance, is being guided by internet videos to exercise safe and beneficial for health? Since it is globally acknowledged that “exercise is medicine”, should we also follow such videos to get our medicines instead of visiting physicians? Moreover, we observe that the popularity of training programs provided by wearables (e.g., global positioning systems, heart rate monitors) increases (Figure 1). However, can such programs replace the individualized “traditional” programs developed by Sports and Health professionals?

Although such questions seem simple, they highlight the need for exercise prescription by Sports and Health professionals especially, in the era of the Covid-19 pandemic, where the access to counseling by professionals is difficult. Maybe it is time for public health-policy makers to encourage people not simply to exercise, but to get guided by Sports and Health professionals to do so.

## Figures and Tables

**Figure 1:**
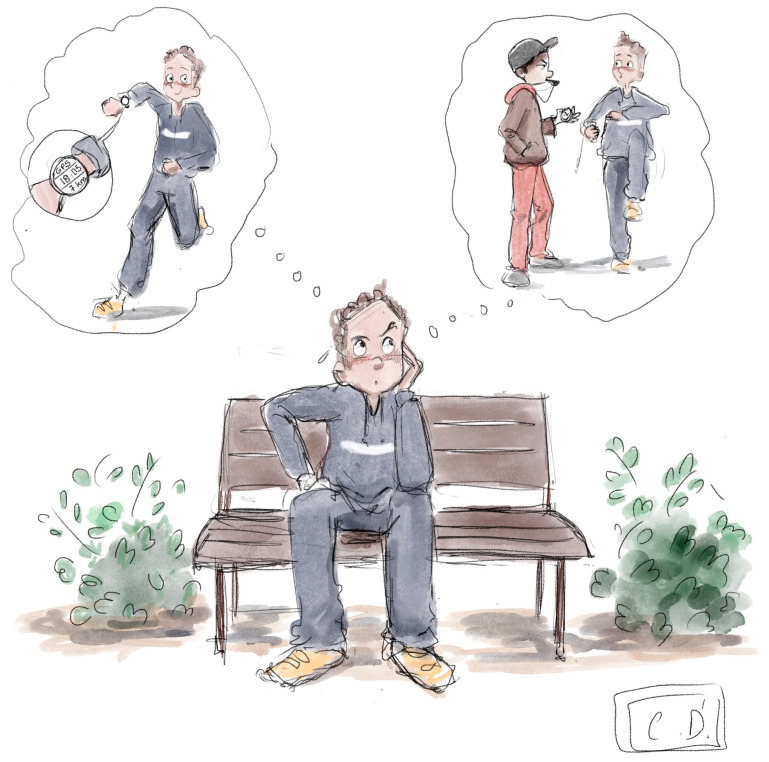
Is it time for Sports and Health in the era of Covid-19 pandemic?
